# Male-Biased microRNA Discovery in the Pea Aphid

**DOI:** 10.3390/insects12060533

**Published:** 2021-06-08

**Authors:** Xiaomi Liu, Erica L. Culbert, Jennifer A. Brisson

**Affiliations:** Department of Biology, University of Rochester, Rochester, NY 14627, USA; eculbert@u.rochester.edu

**Keywords:** microRNA, sex bias, sexual dimorphism, epigenetics, plasticity

## Abstract

**Simple Summary:**

Males and females develop from a genome that is largely similar, yet the sexes have dramatically different traits. How this happens has driven interest in the role of epigenetic mechanisms—i.e., changes in gene function that are not due to changes in the DNA—in regulating sexual dimorphisms. Our focus here is on differences between the sexes in one epigenetic mechanism, the downregulation of gene expression by small RNAs called microRNAs. Typical genomes code for hundreds of microRNAs and each one can target many protein-coding RNAs, ultimately causing them to produce fewer protein copies. Here, we focus on microRNAs in male versus female pea aphids. We use small RNA sequence data to identify all the microRNAs in the pea aphid genome and to find microRNAs that are highly biased towards expression in males. This work is foundational for future studies of the epigenetic basis of sex differences in the pea aphid.

**Abstract:**

Epigenetic mechanisms modulate gene expression levels during development, shaping how a single genome produces a diversity of phenotypes. Here, we begin to explore the epigenetic regulation of sexual dimorphism in pea aphids (*Acyrthosiphon pisum*) by focusing on microRNAs. Previous analyses of microRNAs in aphids have focused solely on females, so we performed deep sequencing of a sample containing early-stage males. We used this sample, plus samples from Genbank, to find 207 novel pea aphid microRNA coding loci. We localized microRNA loci to a chromosome-level assembly of the pea aphid genome and found that those on the X chromosome have lower overall expression compared to those on autosomes. We then identified a set of 19 putative male-biased microRNAs and found them enriched on the X chromosome. Finally, we performed protein-coding RNA-Seq of first instar female and male pea aphids to identify genes with lower expression in males. 10 of these genes were predicted targets of the 19 male-biased microRNAs. Our study provides the most complete set of microRNAs in the pea aphid to date and serves as foundational work for future studies on the epigenetic control of sexual dimorphism.

## 1. Introduction

Epigenetic processes such as DNA methylation, histone modifications, and the deployment of regulatory, noncoding RNAs have long been implicated in controlling insect phenotypic variation (reviewed in [1]). This is most obvious in species that exhibit adaptive phenotypic plasticity (see examples in [2]) in which a single genotype can result in multiple phenotypes depending upon the context of the developmental environment. The resulting phenotypic differences are, by most definitions, epigenetically controlled, given that there are no changes to the underlying DNA sequences.

Like traits that are phenotypically plastic, sexually dimorphic traits are largely mediated by epigenetic processes. This is especially true in insects with no sex chromosomes, such as species that use haplodiploid or XO sex determination. In these species, males and females are genetically identical except for chromosome dosage, meaning that a single genome must epigenetically give rise to male and female phenotypes via sex-specific patterns of gene expression (reviewed in [3]); compare to species with sex chromosomes, where sex can be determined genetically, such as the M factor in *Aedes aegypti* [4]). Sex-specific genome-wide transcriptional profiling has repeatedly revealed large numbers of sex-biased genes across a wide range of taxa [5]. Yet it remains unclear exactly how insect sex-biased transcriptional profiles are programmed epigenetically, although recently multiple studies have explored sex-biased DNA methylation patterns [6,7,8,9].

Here we focus on epigenetic sex differences in the pea aphid (*Acyrthosiphon pisum*). Aphids are known for their summer, all-female asexual phase but they have a lesser-known sexual phase in the fall, wherein males and sexual females are produced and mate. Males are generated from asexual females via loss of one X chromosome [10], so males are XO and females XX. Otherwise, their genomes are identical. Dosage compensation occurs for X-linked genes, based on evidence that male and female expression of X-linked genes is near equal [11].

As in insects generally, much remains to be discovered about the epigenetic basis of sexual dimorphism in aphids. Sex-biased differences in DNA methylation have been observed [6] but no studies to date have examined the role of histone modifications or regulatory RNAs in aphids. Our goal here was to take a first look at sex-biased microRNA expression. MicroRNAs (miRNAs) are a class of small (18–24 nucleotide) non-coding RNAs that regulate gene expression by targeting the 3′UTR of those genes (reviewed in [12]). Targeted mRNAs are subjected to translation inhibition or degradation, resulting in their downregulation [13]. Individual miRNAs can target hundreds of genes for regulation, meaning they have the power to dramatically shape the male or female transcriptome [14,15]. The vast majority of gene expression studies in aphids have focused on asexual females and miRNAs investigations are no exception. MicroRNAs discovery has been performed in multiple aphid species, including the pea aphid (e.g., [16,17,18,19,20]), but never using male samples.

The purpose of our study was threefold. First, we wanted to identify the most complete set of miRNAs in the pea aphid, to serve as a resource for future investigations into how miRNAs regulate sexual dimorphism and phenotypic plasticity in this species. Specifically, we performed deep sequencing of mixed developmental stages of males (embryos through second instar nymphs), since miRNAs had never before been examined in male aphids. Since previous pea aphid miRNAs studies (e.g., [18,20]) had worked with a highly fragmented genome, we also localized putative miRNAs to a recently released, chromosome-level assembly for this species [21]. Second, we wanted to identify miRNAs with male-biased expression and localize them to the X versus the autosomes. Male-biased protein-coding genes are more commonly found on the X chromosome in pea aphids and all protein-coding genes have comparatively lower levels of expression on the X compared to the autosomes [22,23,24] so we tested the hypothesis that miRNAs would exhibit these same patterns. Finally, we wanted to identify possible targets of male-biased miRNAs. We, therefore, sequenced the expressed protein-coding RNAs of male and female first-instar nymphs to find genes that were down-regulated in males relative to females. We used target prediction programs to determine if putative targets of the male-biased miRNAs were enriched in genes that were downregulated in males.

## 2. Materials and Methods

### 2.1. RNA Preparation and Sequencing

We obtained male RNA samples from line 409, which produces wingless males [25]. We induced asexual females to produce males by moving them from a summer to a fall photoperiod. Once an asexual female begins producing males (~three generations later), they continue to produce males until death. We identified females producing males by isolating them onto individual, small Petri dishes and following their offspring until adulthood. Male-producing females were then allowed to lay offspring for several days so that we could collect first instar nymphs (one-day-old) and second instar nymphs (three days old). We also dissected the females to collect embryos. We, therefore, collected samples from three developmental stages: embryos, first instar nymphs, and second instar nymphs. Each sample was a pool of 15–20 individuals. We extracted total RNA separately from each pool using a standard TRizol protocol plus Dnase1 treatment and then pooled them in equal proportions in order to sequence a single sample. Small RNAs were size selected, processed into an Illumina TruSeq Small RNA library, and sequenced using Illumina single-end, 50-nt reads in a single lane by the University of Rochester’s Genomic Center. Small RNA data are available in the NCBI BioProject PRJNA732656.

### 2.2. Novel miRNA Identification

Some 103 mature miRNAs were previously found in the pea aphid genome [20]. We used miRDeep2 to predict novel miRNAs from 50 small RNA-Seq data sets. Some 49 are publicly available data from NCBI (see accession numbers in Supplementary Table S1) and one is a small RNA-Seq data from the males set generated by our lab (as described in the “RNA preparation and sequencing” section). We processed raw reads using Trim Galore (Trim Galore. Available online: https://www.bioinformatics.babraham.ac.uk/projects/trim_galore/ (accessed on 1 June 2021)) and FastQC (FastQC. Available online: https://www.bioinformatics.babraham.ac.uk/projects/fastqc/ (accessed on 1 June 2021)). We trimmed off adaptor sequences, filtered out poor quality sequences (quality score cutoff 20), and discarded sequences shorter than 18 bp or longer than 35bp. All processed reads were combined and used to predict miRNA candidates using miRDeep2 [26]. For all analyses, we used the chromosome-level genome assembly, *Acyrthosiphon pisum* genome v3.0.

Previously known pea aphid miRNAs from miRBase (miRbase. Available online: http://www.mirbase.org/ (accessed on 1 June 2021)) and mature miRNAs from five other species (*Drosophila melanogaster*, *Locusta migratoria*, *Tribolium castaneum*, *Apis mellifera*, *Aedes aegypti*) were used as reference inputs for miRDeep2. MiRDeep2 assigns a score indicating the likelihood that a detected RNA is indeed a mature miRNA based on many features [27]. We filtered the results using the miRDeep2 score ≥1 as cutoff (true positive rate 80 ± 3%, detect rate 91%). All predicted miRNAs that lacked candidate star and loop sequences were also removed. Predicted miRNA precursor sequences were all Blasted [28] against known miRNA precursor sequences from miRBase. Novel miRNAs were included in our set based on homology (if it had a homolog hit) and/or expression (minimum of 30 reads mapped to mature sequence). Otherwise, it was classified as only a miRNA candidate. The expression levels for the predicted mature, star, and loop sequences were also inspected to assess mature versus star strand assignment.

Novel miRNAs were named based on the mature sequence they code for. Different loci coding for the same mature miRNA sequences were assigned the same family number. An “x” was placed in front of the number within the microRNA name to indicate that it was novel. We used the combination of these novel miRNAs and previously described miRNAs for all downstream analyses.

### 2.3. miRNA Distribution Analyses

To analyze the chromosomal distribution of miRNAs, genomic coordinates for previously described miRNA precursors [20] were retrieved by Blasting [28] the 123 precursor sequences against the pea aphid genome, resulting in 135 identified loci. These loci have 100% query coverage and no mismatches. The coordinates for newly predicted miRNA precursors were obtained from the miRDeep2 output. The random distribution of precursor coding loci along chromosomes was tested using the chi-square goodness of fit test. MiRNA clusters were defined as those with neighboring miRNAs separated by less than 10 kb [18]. We also classified clusters into two types: a single-family cluster, which codes for miRNAs that are paralogs of each other; or a multifamily cluster, which codes for miRNAs that are not all paralogs of each other.

### 2.4. miRNA Differential Expression Analyses

To find potential male-biased miRNAs, we compared the expression level of different miRNAs between male and female samples. We used the processed reads from 49 out of the 50 datasets. We excluded one (accession #SRR6981552) from this step because of the lack of sample description and, thus, we didn’t know if it was from females. All novel miRNA precursor sequences and previously described miRNA precursors were used. For any repetitive miRNA precursor sequences, only one record was kept in the combined precursor library as representative. Reads were mapped to the miRNA precursor library using bowtie 1.2.3 with the default setting [29]. Normalized counts in terms of counts per million were generated with customized scripts.

### 2.5. Identification of Potential miRNA Targets

We predicted potential miRNA target sites at all pea aphid mRNA 3′ UTR regions using three programs: PITA [30], RNAhybrid 2.1.2 [31], and RIsearch2 2.1 [32]. For all programs, we set seed length ≥ 7, we allowed no G:U wobble at seed region and no mismatch at seed region was forced to be met either when running the program or manually checked after obtaining the result. The cutoff for the energy threshold for predicted interactions was 0 kcal/mol for PITA and RNAhybrid, and −15 kcal/mol for RIseach to balance the predicted target site number. For RNAhybrid, the extra criterion of *p*-value < 0.05 was used when running the prediction.

### 2.6. RNA-Seq and Differential Expression Analyses

The pea aphid line 409 (the same line used for male small RNA-seq) was used for protein-coding RNA-Seq. First instar wingless males were obtained, pooled, and RNA extracted as above. Three wingless male samples were used for a total of three biological replicates. Female samples were collected from asexual females of the “F1” line, which is the parent of the 409 line. Asexual females were reared on *Vicia faba* plants covered with cages at low density in climate-controlled rooms at approximately 19 °C, 35% humidity, and 16 L:8 D cycle. We moved individual adult females onto single 90mm Petri dishes with a fresh leaf to produce offspring for 24 h and then discarded the adult. First instar wingless females were collected within 24 h. We collected three biological replicates, with each being a pool of 10 to 15 nymphs and with all nymphs coming from a different mother. RNA was extracted as above. Samples were sequenced by NovaSeq paired-end 150nt sequencing. The raw reads data are available at NCBI BioProject PRJNA732656.

We processed raw reads using TrimGalore (Trim Galore. Available online: https://www.bioinformatics.babraham.ac.uk/projects/trim_galore/ (accessed on 1 June 2021)) and FastQC (FasrQC. Available online: https://www.bioinformatics.babraham.ac.uk/projects/fastqc/ (accessed on 1 June 2021)). We trimmed off adaptor sequences, filtered out bad quality sequences (quality score cutoff of 20), and discarded sequences shorter than 20 bp. The filtered sequenced reads were mapped to the reference genome (v2.0) using bowtie2 version 2.3.5.1 [33]. Counts were obtained using featureCounts version 2.0.0 [34]. Differential expression analysis was performed using the DEseq2 R package [35]. Padj < 0.05 and greater than four-fold change was used as a significant cutoff.

## 3. Results

### 3.1. RNA Sequencing

We performed small RNA deep sequencing of a mixed stage male sample (embryos, first instar nymphs, second instar nymphs) to obtain over 96 million raw reads. As is typical for miRNAs [36], after processing, read length varied from 18 to 35 nucleotides, peaking at 22 nucleotides (Figure S1). We then combined these processed reads with processed reads from 49 other pea aphid small RNA libraries (Table S1), totaling almost 252 million reads for downstream analyses. We also sequenced protein-coding RNAs from three male and three female libraries of first instar nymphs, averaging 41 million reads per sample (Table S1).

### 3.2. Identification of Novel miRNAs

Some 103 mature miRNAs were previously identified in the pea aphid genome [20]. Our goal here was to discover novel miRNAs. For the following descriptions, recall that multiple miRNA loci can code for the same precursor miRNA and multiple precursors can be processed into the same mature miRNA. We identified 251 loci encoding 209 novel miRNA precursors at a true positive rate of 80% ± 3% (miRDeep2 score of ≥1; Table S2). After the extra filtering steps described in the methods section, our final set had 207 novel miRNA coding loci, with 167 putative miRNA precursors and 144 mature miRNAs (Table S3). The expression levels for the predicted mature, star, and loop sequences of each is shown in Figure S2. Only three predicted precursors had a higher read count for the star strand than the mature miRNA strand, indicating overall high accuracy of mature and star strand assignment. These three were not filtered out because their mature and star sequence read counts were above our cutoff of 30.

In the course of discovering novel miRNAs, we also looked to see how well our methods performed in terms of the previously discovered miRNAs [20]. Using our initial filtering step of a miRDeep2 score of ≥1, we found 93 of the 103 previously described mature pea aphid miRNAs. With the additional criteria described in the methods, we found 85 of those 93 miRNAs. We, therefore, conclude our miRNA detection methods have a high true positivity rate.

### 3.3. Localization of miRNA Loci to Chromosomes

**Figure 1:**
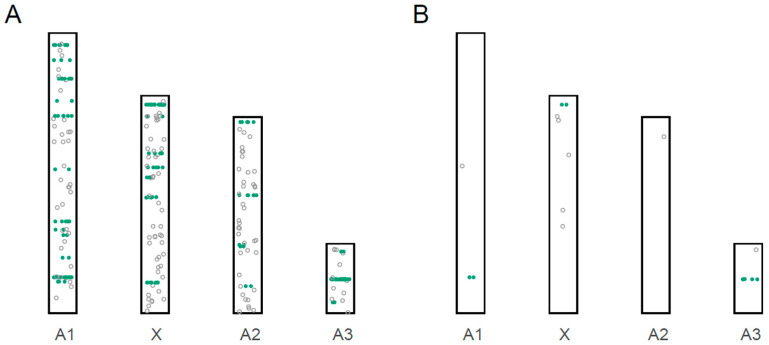
The distribution of miRNA coding loci across the four pea aphid chromosomes. (**A**) The distribution of all miRNA coding loci. (**B**) The distribution of highly male-biased miRNA coding loci (5× higher in males compared to females). Clusters are indicated by green, solid circles, while non-cluster miRNAs are indicated by open circles.

We combined our 207 novel miRNA coding loci described above with the 135 miRNA coding loci previously described [20] for a total of 342 miRNA coding loci. Some 313 of the coding loci are found on one of the pea aphid’s four chromosomes, while 29 are on unplaced genomic scaffolds (Figure 1 and Figure S3, Table 1 and Table S4).

To identify groups of miRNA loci on chromosomes, we defined a miRNA cluster as two or more miRNAs wherein each miRNA was less than 10 kb from the next. These criteria resulted in the identification of 39 clusters, with 184 out of 342 miRNA coding loci (53.8%) arranged in clusters (Figure 1, Table S4). This finding is consistent with previous studies in the pea aphid, which found that over half of the predicted miRNA loci were found in clusters [18,20]. There are two types of clusters: single-family clusters that code for miRNAs that are paralogs of each other, or multifamily clusters that code for miRNAs that are not all paralogs of each other. We found that 12 out of 39 clusters are single-family clusters. The number of coding loci ranges from two to six in single-family clusters, with the longest cluster span through 8052 bp in size. For the 27 multifamily clusters, the number of coding loci ranges from two to 24, with the longest cluster spanning 43,833 bp.

**Figure 2:**
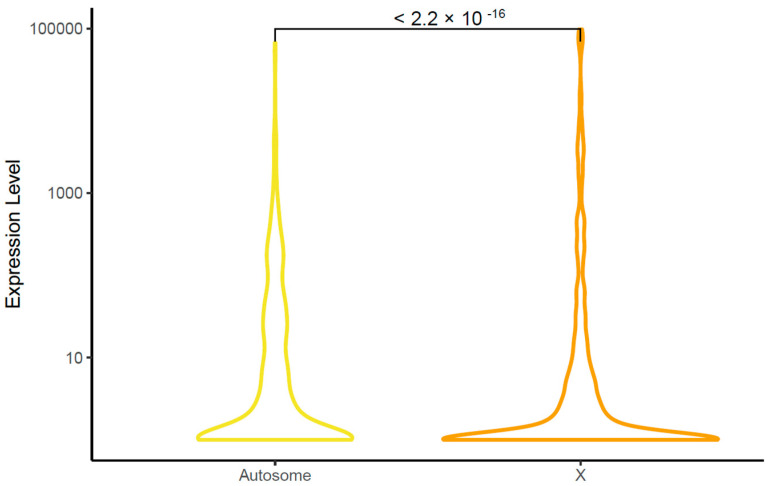
miRNA expression levels on autosomes versus the X chromosome. The expression level is shown on the *y*-axis in terms of log10 (cpm + 1). Colors indicate chromosome type (autosome: yellow, X chromosome: orange).

Given previous reports that protein-coding genes on the autosomes are expressed at higher levels than those on the X chromosome in the pea aphid [23,24], we examined corresponding patterns of miRNA gene expression levels. We found that the mean expression level of miRNA on the X chromosome is significantly lower than on autosome (*p*-value < 2.2 × 10^−16^, Wilcoxon signed-rank test, Figure 2).

### 3.4. Male-Biased miRNAs

**Figure 3:**
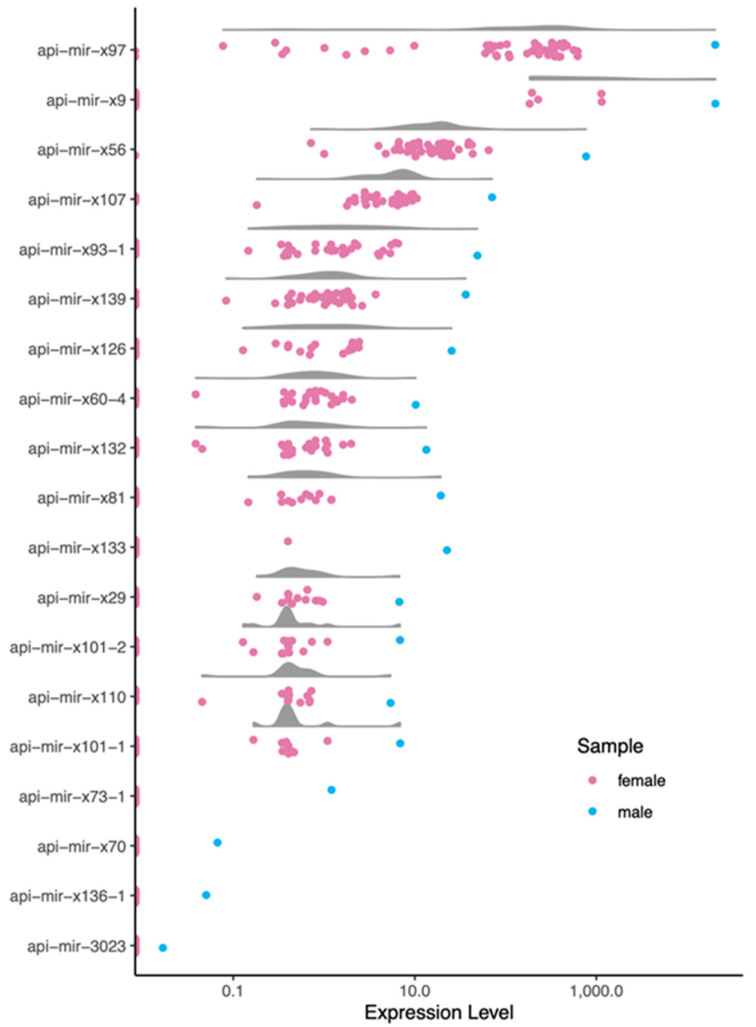
Expression level of male-biased miRNAs across libraries. Expression level is shown on the *x*-axis in terms of log (cpm). Individual data points from female (pink) or male (blue) samples are shown. A raincloud plot summarizes the expressed data points (gray cloud) but excludes the unexpressed data points (left along *y*-axis).

Our next goal was to identify male-biased miRNAs. We calculated miRNA expression levels in terms of counts per million (cpm) across all samples (Table S5). Because we only had a single sample derived from male pea aphids (all others are from females), we conservatively called a miRNA male-biased if its expression was five times higher in the male sample compared to the highest expression in one of the 48 female samples. This resulted in a set of 19 putatively male-biased miRNAs (Table 1 and Table S6, Figure 3). Although a higher proportion of male-biased miRNAs were located on the X-chromosome compared to non-biased miRNAs (41% versus 32%; Figure 1B), the distribution across the chromosomes was not significantly different (X^2^ contingency text, X^2^ = 6.8, *p*-value > 0.05; unplaced genomic scaffolds excluded). Because of the low sample size of male-biased miRNAs, we then used a less stringent cutoff of two times higher in male samples, resulting in the identification of a total of 25 male-biased microRNAs across the four chromosomes (Table S6). The distribution for this set was not statistically equal across the chromosomes (X^2^ contingency text, X^2^ = 10.4, *p*-value < 0.025; unplaced genomic scaffolds excluded), with higher proportions than expected on both the X chromosome and on chromosome A3. The overrepresentation of male-biased genes on the X, in particular, has previously been observed with protein-coding male-biased pea aphid genes [23,24].

### 3.5. Predicted Targets of Male-Biased miRNAs

**Figure 4:**
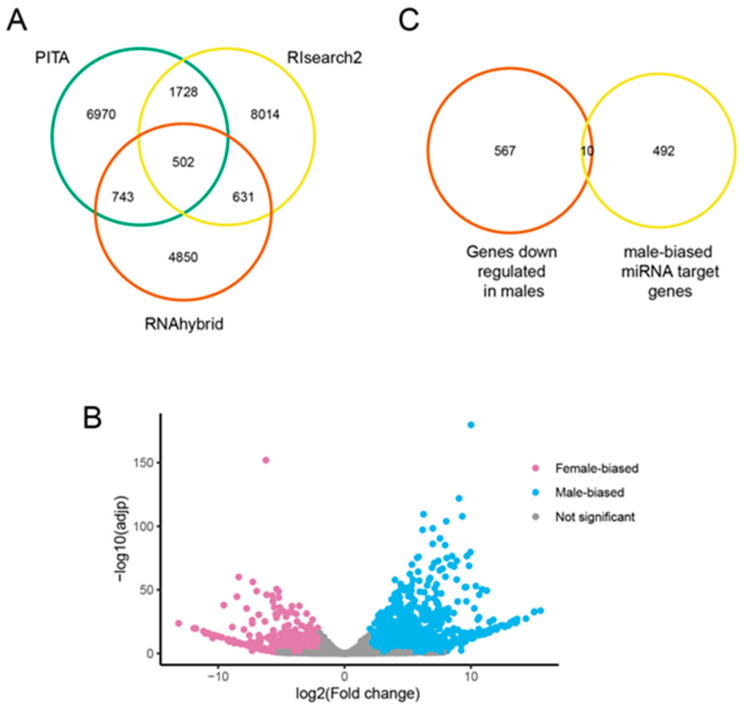
Male-biased miRNA targets. (**A**) Each circle shows the number of possible target genes from each of the three target prediction programs (PITA, Rlsearch2, and RNAhybrid). (**B**) Results of the RNA-seq analysis. The *x*-axis represents the log2 fold change of each expressed gene in the aphid genome. The *y*-axis shows the statistical significance of differential gene expression (−log10 adjpval) for each gene. Genes that were differentially expressed at a false discovery rate (FDR) of less than 0.05 and change by greater than 4 fold are shown with pink for female-biased genes and blue for male-biased genes. (**C**) Venn diagram comparing the miRNA predicted target with genes down-regulated in males.

We predicted the genes possibly targeted by the 19 most strongly (greater than fivefold higher) male-biased miRNAs using three different programs: PITA [30], RNAhybrid 2.1.2 [31], and RIsearch2 2.1 [32]. Each predicted thousands of targets but the intersection of all three produced a list of 502 putative targets (Figure 4A). Many miRNAs target multiple genes so we ensured that the same miRNA-target interaction relationship existed to call a gene in the overlap list.

To determine if the possible male-biased miRNA targets were among genes with different expression levels between males and females, we performed protein-coding RNA-Seq using three replicates of males and females each for first-instar nymphs. Of the 23,219 expressed genes, 2678 were significantly differentially expressed (padj < 0.05, fold change > 4, Table S7). Of those, 577 had higher expression in females and 2101 had higher expression in males (Figure 4B). Of the 577 genes that were at lower expression males, 10 genes were predicted male-biased miRNA targets (Figure 4C, Table S8).

## 4. Discussion

Here we present the most complete set of miRNAs ever discovered in the pea aphid. MiRNAs can have sex-biased expression, as demonstrated across a range of organisms [37,38,39]. Therefore, the previous lack of miRNA libraries derived from males served to underestimate the total miRNAs in pea aphids. We have nearly doubled the number of miRNAs described by using a sample collected from early stages of male development. While some of these are possible false positives, the number brings it up closer to the numbers in more well-studied species like *Drosophila melanogaster* or *Caenorhabditis elegans* where 469 and 437 miRNAs are known, respectively [30,40]. Future studies incorporating later stages of male development are likely to reveal even more miRNA loci, especially given that often many male-biased miRNAs are highly expressed in male testes [37,41].

Given our use of the chromosomal-level pea aphid genome assembly [21], we were able to investigate miRNA gene location and expression levels on the X chromosome versus the autosomes. Evolutionary forces can differently affect the sex chromosome compared to the autosomes, shaping gene content on each [42]. Aphids have an unusual life cycle that is dominated by asexual generations. The effective population sizes of the X and autosomes are therefore equal [43], unlike in other, sexual XO systems where the effective population size of the autosome is larger than that of the X chromosome. Moreover, the relative rarity of sexual morphs (females and males) results in reduced purifying selection on genes with morph-biased expression [22,44]. Likely due to these factors, Li et al. [24] and Jaquiery et al. [23] found male-biased protein-coding genes enriched on the X chromosome. We, too, found a pattern of miRNA loci with male-biased expression being enriched on the X chromosome. These same previous studies [23,24] also found a generally lower expression level for X-linked protein-coding genes compared to autosomal protein-coding genes, regardless of whether or not the gene was expressed in a sex-specific manner. We observed the same pattern with miRNA loci. These same forces, therefore, affect the genomic distribution of miRNA loci as well as protein-coding loci.

Our study also predicted possible targets of male-biased miRNAs among protein-coding genes with lower expression in males compared to females. Our goal was to use conservative criteria to obtain a reliable list of targets, by using three prediction programs for target identification and by only considering protein-coding genes differentially expressed if they had a multiple comparison-corrected *p*-value < 0.05 and a fold change of four or over. Additional possible miRNA/target gene interactions could likely be found by loosening these criteria. Of the ten genes we identified, one (ACYPI31408) is of particular interest. It is a predicted UDP-glucoronosyltransferase/glycosol transferase (UGT). UGTs are a family of enzymes that play a variety of roles in the breakdown of external and endogenous compounds. In insects, their roles in detoxifying plant defenses [45] and in olfaction are especially well studied [46]. Multiple UGTs have sex-biased expression in *Drosophila* [47], including one that has to be downregulated in male antennae to play its role in sex pheromone sensing [48]. Future studies could use dual-luciferase reporter assays to test for binding between the api-mir-3023 miRNA and this gene. The other nine genes code for a vesicular glutamate transporter (ACYPI065144) and eight uncharacterized proteins.

A larger defined set of miRNAs also sets the pea aphid up for future studies of miRNA control of phenotypic plasticity, both in terms of the sexual versus asexual plasticity and the wing versus wingless plasticity. Comparisons between morphs within each polyphenism are associated with a wealth of gene expression differences [49,50,51,52], which are undoubtedly responding to epigenetic regulation [53,54]. Indeed, a previous study in the brown citrus aphid (*Aphis citricidus*) showed control of its wing plasticity via a miRNA, miR-9b [17]. Profiling of sexual versus asexual female morphs in the pea aphid, as well as winged versus wingless morphs of the English grain aphid (*Sitobion avenae*) [16] and the planthopper (*Nilaparvata lugens*) [55], showed many miRNA expression level differences between morphs of each comparison. Epigenetic investigations of these and other insect plasticities are likely a rich source of future studies.

## 5. Conclusions

Here we have identified the most complete set of pea aphid miRNAs to date and localized them to a chromosome-level assembly of the pea aphid genome. We find lower miRNA expression levels on the X and enrichment of male-biased miRNAs on the X, similar to patterns observed for protein-coding genes, suggesting similar evolutionary forces are acting on miRNA loci and protein-coding loci. Our analysis of differential protein-coding gene expression between first instar males and females also provides a wealth of data for future investigations of early sexual differentiation.

## Figures and Tables

**Table 1:** miRNA coding loci distribution summary.

Chromosome/ Scaffold	Size of Scaffold (bp)	Proportion of Genome	# of Novel miRNAs	# of Previously Described miRNAs	Total # of miRNA	# of Highly Male-Biased miRNAs (5× Higher)
A1	170,740,645	0.32	48	51	99	3
A2	119,541,763	0.22	20	37	57	1
A3	42,333,646	0.08	45	9	54	6
X	132,544,852	0.24	72	31	103	7
Unplaced scaffolds	75,959,697	0.14	22	7	29	2
Total	541,120,603	1	207	135	342	19

## Data Availability

Accessions used in this study are listed in Table S1. All raw reads data are available at NCBI BioProject PRJNA732656.

## Supplementary Materials

The following are available online at https://www.mdpi.com/article/10.3390/insects12060533/s1, Figure S1. Length distribution of reads from the deep sequencing of male small RNAs. Figure S2. miRNA precursor region-specific expression level patterns. Figure S3. The distribution of miRNA coding loci across the pea aphid unplaced genomic scaffolds (UGS; those not included on the four major chromosomes). Clusters are indicated by green, solid circles, while non-cluster miRNAs are indicated by open circles. Figure S4. The distribution of male-biased miRNA coding loci across the pea aphid unplaced genomic scaffolds (UGS; those not included on the four major chromosomes). Clusters are indicated by green, solid circles, while non-cluster miRNAs are indicated by open circles. Table S1. Information about RNA-Seq libraries used in this study. Table S2. Survey of miRDeep2 performance for score cut-offs −10 to 10. Table S3. Novel miRNA identification results. Table S4. Location of all miRNAs. Table S5. miRNA expression levels across all samples in counts per million (cpm). Table S6. Differentially expressed miRNAs between females and males. Table S7: Differentially expressed genes between males and females (adjpval < 0.05, log2fold change > 2). Table S8. Possible targets of male-biased miRNAs.
