# Supplementary figures and images for: Male-Biased microRNA Discovery in the Pea Aphid
Source: Insects. 2021 Jun 8;12(6):533. doi: 10.3390/insects12060533 (PMC8228725; doi:10.3390/insects12060533)

Figure S1

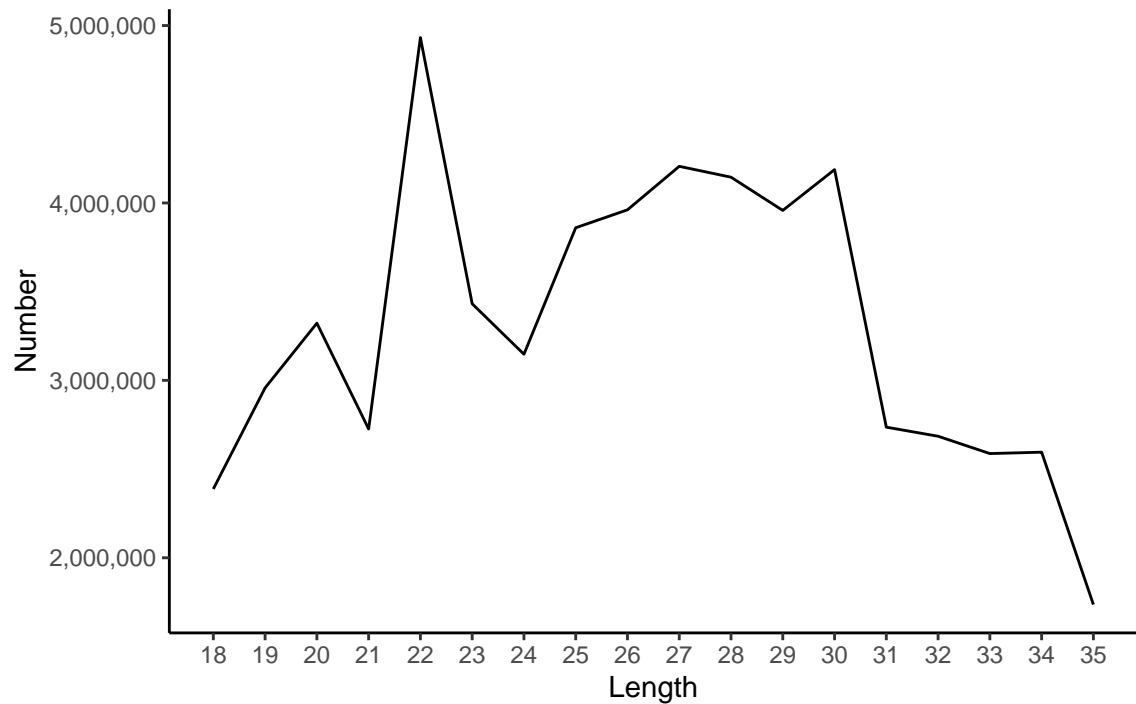

Supplement: Supplementary file 1 [file insects-12-00533-s001.zip › supplementary_data/supplementary figure/FigS1.pdf]

Figure S2

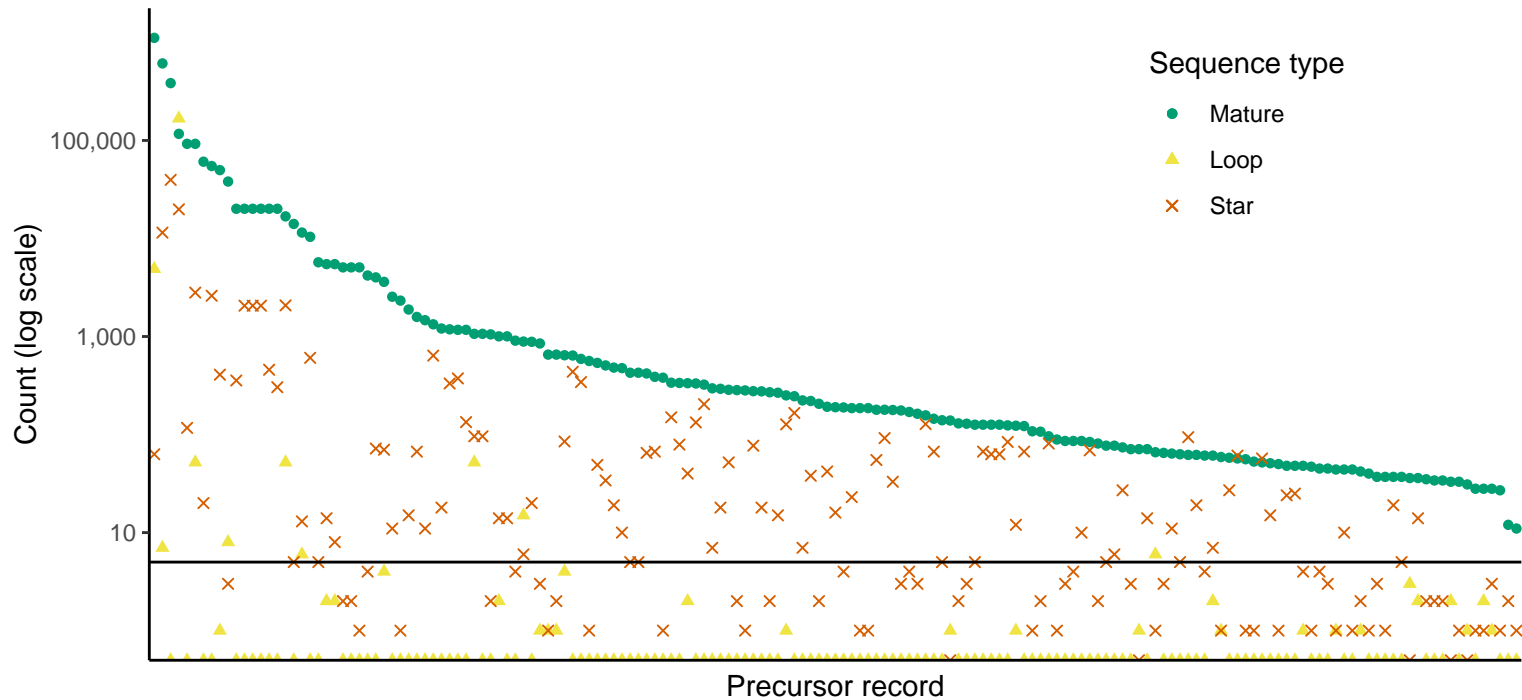

Supplement: Supplementary file 1 [file insects-12-00533-s001.zip › supplementary_data/supplementary figure/FigS2.pdf]

Figure S3

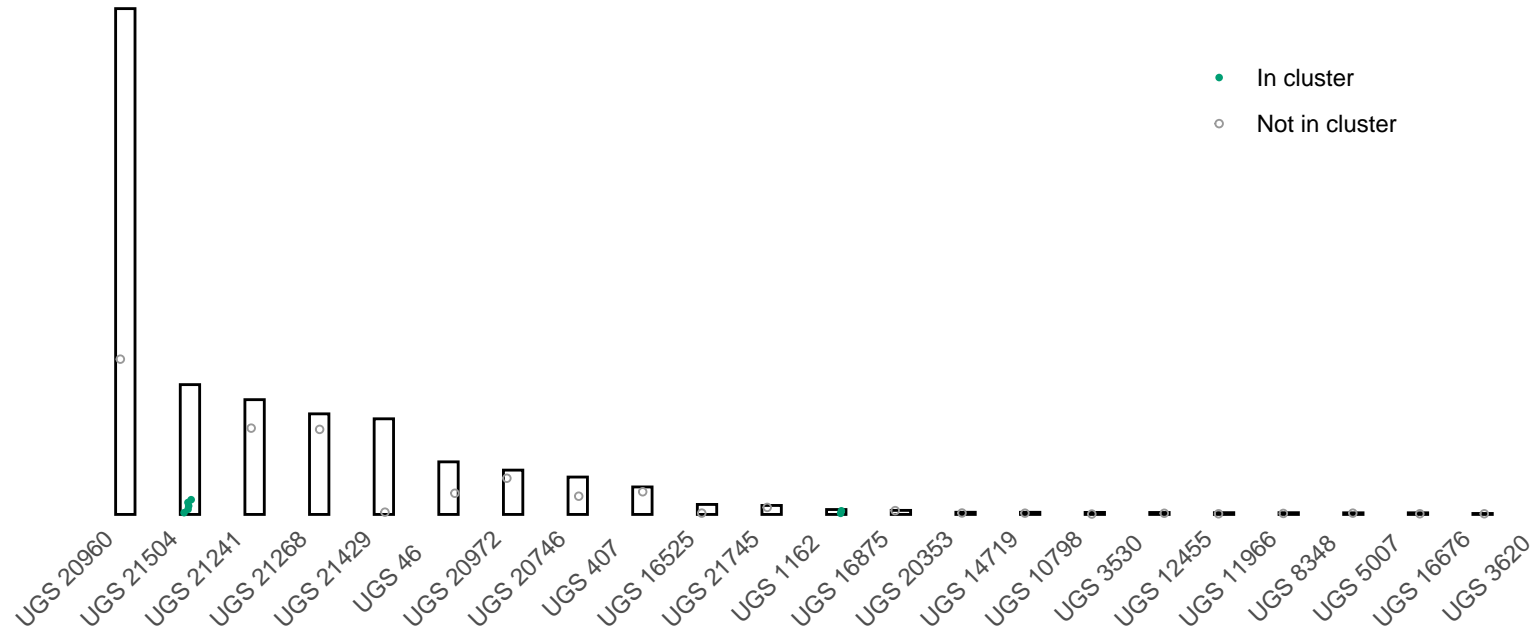

Supplement: Supplementary file 1 [file insects-12-00533-s001.zip › supplementary_data/supplementary figure/FigS3.pdf]

Figure S4

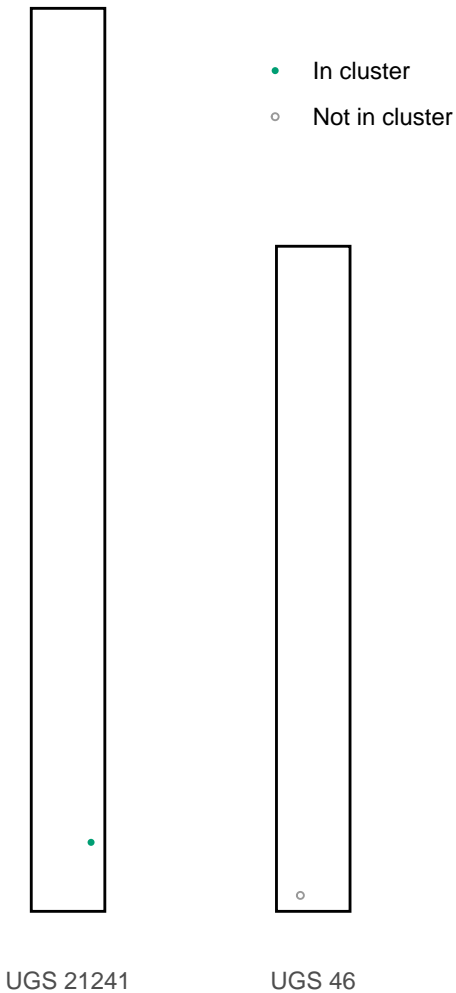

Supplement: Supplementary file 1 [file insects-12-00533-s001.zip › supplementary_data/supplementary figure/FigS4.pdf]
